# Mortality and associated factors among patients with TB-HIV co-infection in Ethiopia: a systematic review and meta-analysis

**DOI:** 10.1186/s12879-024-09683-5

**Published:** 2024-08-02

**Authors:** Sisay Moges, Bereket Abrham Lajore

**Affiliations:** Department of Family Health, Hosanna College of Health Science, Hosanna, Ethiopia

**Keywords:** Tuberculosis, HIV, Mortality, TB co-infection, Ethiopia, Systematic review, Meta-analysis

## Abstract

**Background:**

Tuberculosis (TB) and human immunodeficiency virus (HIV) co-infection is a major public health problem in Ethiopia. Patients with TB-HIV co-infection have significantly higher mortality rates compared to those with TB or HIV mono-infection. This systematic review and meta-analysis aim to summarize the evidence on mortality and associated factors among patients with TB-HIV co-infection in Ethiopia.

**Methods:**

Comprehensive searches were conducted in multiple electronic databases (PubMed/MEDLINE, Embase, CINAHL, Web of Science) for observational studies published between January 2000 and present, reporting mortality rates among TB/HIV co-infected individuals. Two reviewers performed study selection, data extraction, and quality assessment independently. Random-effects meta-analysis was used to pool mortality estimates, and heterogeneity was assessed using I² statistics. Subgroup analyses and meta-regression were performed to explore potential sources of heterogeneity.

**Results:**

185 articles were retrieved with 20 studies included in the final analysis involving 8,113 participants. The pooled mortality prevalence was 16.65% (95% CI 12.57%-19.65%) with I^2^ : 95.98% & p-value < 0.00. Factors significantly associated with increased mortality included: older age above 44 years (HR: 1.82; 95% CI: 1.31–2.52), ambulatory(HR: 1.64; 95% CI: 1.23–2.18) and bedridden functional status(HR: 2.75; 95% CI: 2.01–3.75), extra-pulmonary Tuberculosis (ETB) (HR: 2.34; 95% CI: 1.76–3.10), advanced WHO stage III (HR: 1.76; 95% CI: 1.22–2.38) and WHO stage IV (HR: 2.17; 95% CI:1.41–3.34), opportunistic infections (HR: 1.75; 95% CI: 1.30–2.34), low CD4 count of < 50 cells/mm^3^ (HR: 3.37; 95% CI: 2.18–5.22) and lack of co-trimoxazole prophylaxis (HR: 2.15; 95% CI: 1.73–2.65).

**Conclusions:**

TB/HIV co-infected patients in Ethiopia experience unacceptably high mortality, driven by clinical markers of advanced immunosuppression. Early screening, timely treatment initiation, optimizing preventive therapies, and comprehensive management of comorbidities are imperative to improve outcomes in this vulnerable population.

## Background

The global impact of human immunodeficiency virus (HIV) and TB co-infection on mortality is substantial and tuberculosis (TB) remains the leading cause of death among people living with HIV, accounting for approximately 30% of deaths among HIV-positive individual [1]. In 2018, 21% of the 1.2 million TB deaths occurred in people living with HIV/AIDS [2]. Moreover, TB is recognized as the leading cause of death from infectious diseases globally, resulting in around 214,000 deaths among HIV-positive individuals [3]. The significant mortality rates among TB/HIV co-infected patients highlight the urgent need for effective interventions and strategies to improve outcomes in this vulnerable population. Tuberculosis (TB) and HIV co-infection is a persistent public health crisis in Sub-Saharan Africa (SSA), bearing a disproportionately high burden of disease compared to other regions [4].

In Ethiopia, the burden of TB and HIV co-infection remains a significant public health challenge [5]. Despite the availability of TB and HIV treatment, the mortality rate among TB-HIV co-infected patients in Ethiopia remains high [5–7]. There is a need to identify the factors associated with mortality among TB-HIV co-infected patients in Ethiopia to inform the development of targeted interventions and improve patient outcomes. Patients with TB-HIV co-infection have significantly higher mortality compared to those with TB or HIV mono-infection. For example, mortality during TB treatment in co-infected patients ranges from 16 to 35%, compared to 4–9% in HIV-negative TB patients [8–10]. The risk of mortality starts high during the initial months of TB treatment and remains elevated for at least 18 months [11, 12]. Studies reported mixed death rate and its determinants, study done at Addis Ababa [13] reported the lowest mortality rate of 4.45% mortality while the highest 25.73% was reported from Tigray region [14] and 35.5% in Amhara region [15] in Ethiopia. Moreover, determinants of mortality were also reported include low CD4 cell count, advanced HIV disease, lack of antiretroviral therapy (ART), TB drug resistance, disseminated TB, and other opportunistic co-infections [8, 11, 16–19].

Despite the fact that several individual studies on the mortality and predictors among TB/HIV co-infected patients in Ethiopian have been conducted, this compressive analysis was conducted to estimate the pooled mortality estimate and its predictors among TB/HIV co-infected patients due to the presence of conflicting (inconsistent) findings in primary studies, which makes programmers, policymakers, and healthcare professionals difficult since no evidence has been published in this regard thus far. The findings of this systematic review and meta-analysis will provide valuable information for policymakers, healthcare providers, and researchers to develop targeted interventions and improve patient outcomes among TB-HIV co-infected patients in Ethiopia.

## Methods

This systematic review and meta-analysis is being reported in accordance with the reporting guidance provided in the Preferred Reporting Items for Systematic Reviews and Meta-Analyses Protocols (PRISMA-P) statement [20].

### Eligibility criteria

Studies were included based on predefined criteria regarding population, comparators, outcomes, study design, settings, period, and language. The target population comprised adult patients with TB/HIV co-infection, while studies focusing on specialized sub-populations, such as children, prisoners, or miners, were excluded. For studies evaluating risk factors, the comparison group was TB-HIV co-infected patients without the risk factor of interest. The primary outcome assessed was mortality, reported as the number of deaths, prevalence, or mortality rate among TB/HIV co-infected patients. Eligible study designs included retrospective cohort studies, and cross-sectional studies. Studies involving either TB or HIV mortality alone, single case reports, case series, reviews, commentaries, editorials, and studies not reporting primary outcome were excluded. Only studies conducted in Ethiopia between 2000 and the present and published in English were considered. Additionally, studies that remained unavailable after two email communications with the primary or corresponding author were also excluded from the review. The predefined inclusion and exclusion criteria aimed to identify relevant and high-quality studies focused on the mortality burden and risk factors among adult TB/HIV co-infected patients within the Ethiopian context.

### Data sources and search strategy

A comprehensive systematic search was conducted across multiple electronic databases, including PubMed/MEDLINE, CINAHL, Embase, Google Scholar, and Science Direct, to identify relevant studies on TB/HIV co-infection mortality. Additionally, reference lists of eligible studies were manually searched for potentially relevant articles. The search was performed independently by two authors (SM and BAL) using the following keywords: Tuberculosis OR TB; HIV OR AIDS; Co-infection OR Coinfection; Mortality OR Death OR Survival; Ethiopia OR Ethiopian; Risk factors OR Associated factors OR Predictors; Treatment outcome; Antiretroviral therapy OR ART. Additionally, include methodological terms such as cross-sectional study; Cohort study; Retrospective study; and Prospective study. Combine these keywords using Boolean operators (AND, OR) to create comprehensive search strings that will capture relevant studies across various databases. For example, the following search strategy was employed in the PubMed database as an example: (((“Tuberculosis“[Mesh] OR TB[Text Word] OR tuberculosis[Text Word]) AND (“HIV Infections“[Mesh] OR HIV [Text Word] OR “human immunodeficiency virus” [Text Word])) OR (“Coinfection“[Mesh] OR co-infection [Text Word] OR “multiple infection” [Text Word])) AND (“Mortality“[Mesh] OR mortality[Text Word] OR death [Text Word] OR fatal * [Text Word]). This search combined relevant MeSH terms and free-text words to capture studies on tuberculosis, HIV infections, co-infection, and mortality outcomes. The retrieved studies were imported and managed using EndNote XX reference management software. The search was conducted between January 25, 2023, and December 12, 2024, to identify potentially eligible studies for the systematic review and meta-analysis.

### Screening and selection process

The screening and selection of studies followed a systematic and rigorous approach. Two independent reviewers screened the titles and abstracts of all records retrieved from the database and manual searches against the predefined eligibility criteria. All identified articles were imported into the EndNote XX library, and duplicate entries were removed. After deduplication, two authors independently extracted data from the remaining articles using a Microsoft Excel 2021 spreadsheet. During the extraction process, the title, abstract, and full text of each article were carefully checked to ensure compliance with the inclusion and exclusion criteria established for the systematic review. The information extracted from eligible studies included study characteristics such as the first author’s name, study area, region, year of publication, study period, study design, study participants, sample size, and the number of events (deaths). Any discrepancies or disagreements in data extraction between the two reviewers were resolved through discussion and consensus. To ensure consistency in the data extraction process, both reviewers independently extracted data from the first three eligible articles. Subsequently, they met to compare results, clarify any discrepancies, and refine the abstraction form if necessary. After this initial calibration, one reviewer abstracted data from the remaining articles, and the second reviewer verified the extracted information.

### Risk of bias assessment

The integrity and potential bias of the studies included in our review were meticulously evaluated by two independent reviewers utilizing the Newcastle-Ottawa Scale (NOS) [21] for observational studies. The NOS assesses the risk of bias in three domains, including the selection of the study groups, the comparability of the groups, and the ascertainment of the outcome of interest. The NOS framework allows for a comprehensive assessment, allocating up to four stars for ‘Selection,’ two for ‘Comparability,’ and three for ‘Outcome’ categories.

### Data extraction

A standardized Microsoft Excel spreadsheet was used for data extraction. Two authors (SM and BAL) independently extracted data from the included studies using a predefined checklist. Disagreements were resolved through discussion. The following data were extracted from each primary study: first author’s name, publication year, follow-up duration, magnitude of mortality among TB/HIV co-infected patients with 95% confidence intervals, study region, study setting (hospital, health center, or both), study design, and sample size.

### Outcome variable and measures

The primary outcome of interest in this systematic review and meta-analysis was mortality among TB/HIV co-infected individuals. Mortality was estimated as a percentage, using the number of deaths and the total sample size reported in each individual study. The 95% confidence intervals (CIs) for the mortality estimates were also extracted or calculated from the available data. A random-effects model was employed to pool the mortality estimates across studies, accounting for potential heterogeneity. Additionally, the second outcome of interest was the identification of potential predictors of mortality among TB/HIV co-infected patients. To facilitate this analysis, the effect sizes reported in the primary studies were first transformed into a common metric. Specifically, if hazard ratios (HRs) were reported, they were transformed into log hazard ratios (logHRs) using the natural logarithm function. The standard errors of the logHRs were then computed to obtain the corresponding variance estimates.

### Data synthesis and analysis

A narrative synthesis summarized the study characteristics, populations, interventions, outcome assessments, and key findings. Meta-analysis was conducted using Stata software version 17 to estimate the overall magnitude of mortality and risk factors among TB/HIV co-infected patients. The pooled mortality prevalence and hazard ratios (HRs) with 95% confidence intervals were computed. HRs from primary studies were log-transformed (logHR) to obtain the effect sizes and corresponding standard errors. Forest plots visually displayed the pooled estimates. Heterogeneity was assessed using Cochran’s Q statistic, I^2 statistic, and chi-square test (*p* < 0.05). Heterogeneity levels were classified as low (I^2^: 0–25%), moderate (I^2^: 25–50%), or high (I^2^: ≥50%) [22, 23]. Subgroup analyses and meta-regression explored potential sources of heterogeneity, considering study setting, region, design, publication year, and sample size as covariates. Publication bias was evaluated using Begg’s test and Egger’s test, which quantify funnel plot asymmetry [24, 25]. The report is presented using texts, tables and figures.

## Result

### Selection of studies

**Fig. 1:**
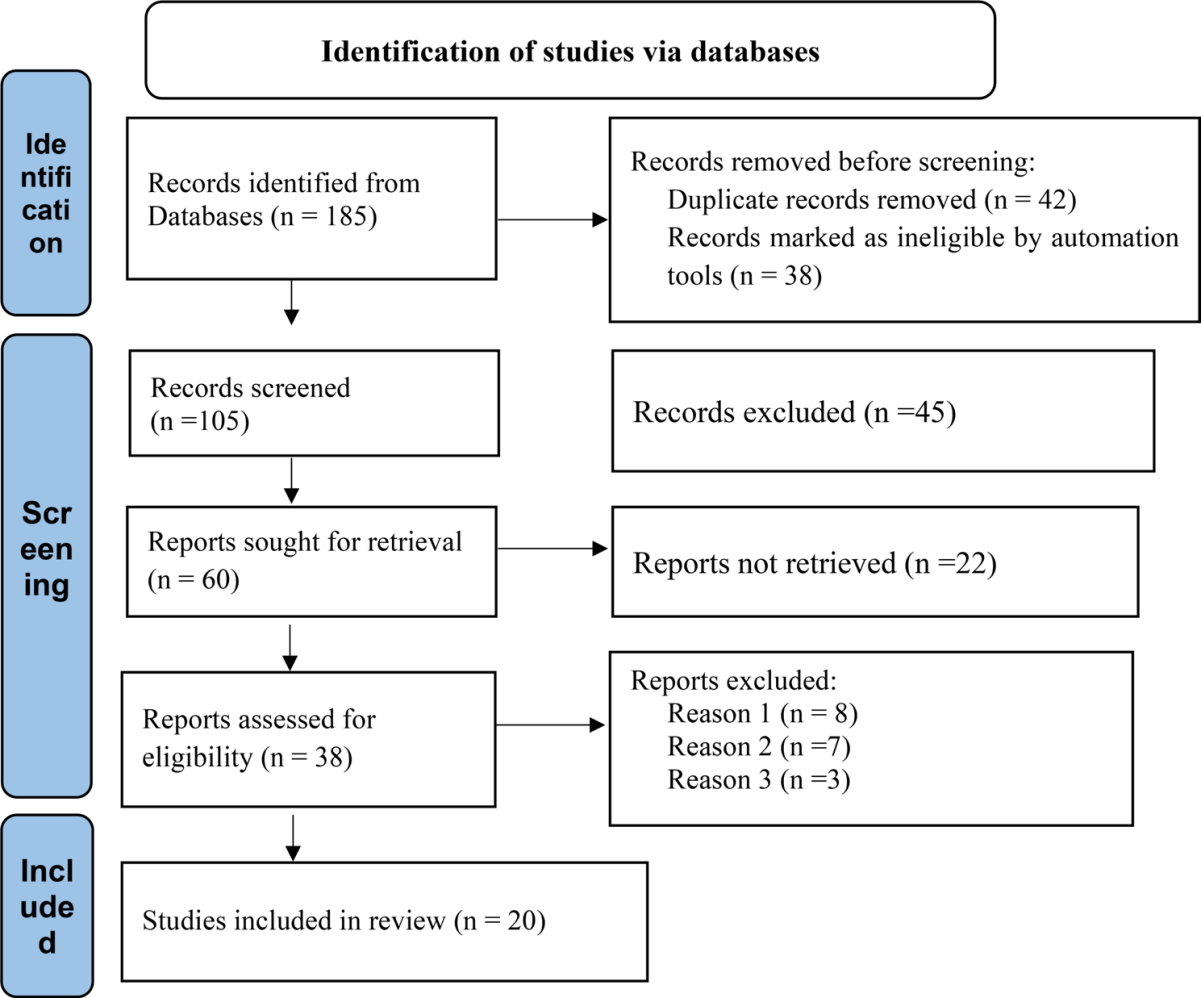
Selection of studies of PRISMA flow diagram

A comprehensive literature search of the database yielded 185 published articles. Of these, 85 articles were retrieved from PubMed, from 62 Google Scholar, and 38 from other sources (Scopus, Web of science, EMBASE, and other individual publications). After assessing titles and removing duplicate publications, 80 articles were excluded, and 105 Records screened abstracts and 45 were removed for not meeting the inclusion criteria. The full texts of 38 articles were assessed for eligibility. Studies were included if they: (1) evaluated the mortality/ death rate, (2) had a study population TB/HIV co-infected, (3) reported number of death, prevalence of death, death rate. Following the full-text review, 20 articles fulfilled the eligibility criteria and were included for final qualitative and quantitative analysis. Reasons for exclusion were; no primary outcome report (*n* = 8), outcomes not clearly indicated (*n* = 7), and lack of extractable data (not empirical) (*n* = 3). (Fig. 1). The selection process is outlined via a PRISMA flow diagram [20]. Overall, 20 studies were identified and included in this systematic review and meta-analysis examining the magintude of mortality among TB/HIV co-infected patients was examined.

### Characteristics of studies

This systematic review included 20 studies conducted in various regions of Ethiopia between 2000 and present involving 8,113 patients. The majority (17 studies) employed a retrospective cohort design, while three were cross-sectional studies [14, 26, 27]. In terms of setting, fourteen studies were carried out in hospitals, two in health centers [28, 29], and four included both hospitals and health centers [13, 19, 29, 30]. The studies predominantly focused on major cities and regional states like Addis Ababa (3 studies) [13, 28, 31], Dire Dawa (2 studies) [19, 32], Tigray (3 studies) [14, 33, 34], Southern Ethiopia (4 studies) [26, 27, 29, 35], Amhara (2 studies) [15, 30], Oromia (4 studies) [17, 18, 36, 37], and Harari (2 studies) [38, 39]. Sample sizes ranged from 188 [26] to 1,123 [13] TB/HIV co-infected individuals across the different studies. The number of recorded deaths spanned from 22 to 93, reflecting the variation in mortality estimates reported. Overall, this review synthesized evidence from studies across various settings and regions within Ethiopia over the past couple of decades (Table 1).

**Table 1:** Characteristics of studies included in the review

Authors	Publication year	Study period	Study region	Study setting	Study design	No. of death	Sample size
Gebre-Mariam [28]	2009	2006–2007	Addis Ababa	Health Center	Retrospective Cohort	31	248
Sime et al. [32]	2022	2014–2016	Dire Dawa	Hospitals	Retrospective Cohort	76	566
Teklu et al. [31]	2017	2005–2015	Addis Ababa	Hospitals	Retrospective Cohort	55	355
Seyoum et al. [13]	2022	2011–2018	Addis Ababa	Hospital & Health center	Retrospective Cohort	50	1123
Teshome Kefale & Anagaw, [26]	2017	2012–2015	Southern Ethiopia	Hospitals	Cross sectional	39	188
Misgina et al. [34]	2019	2010–2016	Tigray	Hospital	Retrospective Cohort	37	295
Sileshi et al. [30])	2013	2009–2012	Amhara	Hospital & Health center	Retrospective Cohort	93	422
Gesesew et al. [18]	2016	2010–2012	Oromia	Hospital	Retrospective Cohort	55	272
Habtegiorgis et al. [19]	2018	2012–2016	Dire Dawa	Hospital & Health center	Retrospective Cohort	79	471
Birhan et al. [15]	2021	2014–2019	Amhara	Hospital	Retrospective Cohort	87	243
Refera & Wencheko, [37]	2013	2006–2011	Oromia	Hospital	Retrospective Cohort	79	502
Lelisho et al. 2022) [36]	2022	2014–2022	Oromia	hospital	Retrospective Cohort	84	402
Wondimu et al. [35]	2019	2007–2017	Southern Ethiopia	hospital	Retrospective Cohort	83	364
Belayneh et al. [14]	2015	2009–2011	Tigray	hospital	Cross sectional	88	342
Weldegebreal et al. [39]	2018	2008–2014	Harari	Hospital	Retrospective Cohort	54	627
Tola et al. [38]	2019	2012–2017	Harari	hospital	Retrospective Cohort	27	349
Shaweno & Worku, [29]	2012	2006–2010	Southern Ethiopia	Health Center	Retrospective Cohort	50	370
Fiseha et al. [27]	2015	2010–2013	Southern Ethiopia	hospital	Cross sectional	22	397
Abrha et al. [17]	2015	2010–2012	Oromia	Hospital	Retrospective Cohort	55	272
Gezae et al. [33]	2020	2015–2018	Tigray	Hospital	Retrospective Cohort	70	305

### Risk of bias assessment

The risk of bias and quality of studies included in this review were meticulously evaluated by two independent reviewers utilizing the Newcastle-Ottawa Scale (NOS) [21] for observational studies. The NOS assesses the risk of bias in three domains, including the selection of the study groups, the comparability of the groups, and the ascertainment of the outcome of interest. Based on the NOS framework within the ‘Selection’ domain, eight studies [13, 15, 18, 19, 30–32, 34] achieved the maximum score of four stars, indicating lower risk of selection bias since the studies indicated clear inclusion and exclusion criteria and encompassed all TB/HIV co-infected individuals. However, six studies [26, 28, 35, 37–39] were assigned a score of three stars in this category due to their potential selection bias without clear eligibility criteria in their studies, which may affect the representativeness of the study population. For ‘Comparability,’ eleven studies [13, 14, 19, 27, 29–35, 38] were awarded two stars, indicating a robust control for potential confounders within their design or analysis, thereby reducing the likelihood of confounding bias. The other nine studies received one star, suggesting a possible presence of confounding factors that were not adequately addressed. In the ‘Outcome’ category, the majority of studies were given three stars, denoting a high-quality approach to ascertaining the outcomes of interest, specifically mortality among TB/HIV co-infected individuals. Nonetheless, three studies [14, 26, 27] received a score of two stars due to their cross-sectional design, which may lead to potential outcome measurement bias, and may introduce some limitations in terms of establishing temporal relationships between risk factors and outcomes. Overall, the studies’ total scores ranged from 7 to 9 out of a possible 9, signifying that the body of observational studies we considered was generally of high quality, with a minimal risk of bias present.

### Pooled magnitude of mortality

**Fig. 2:**
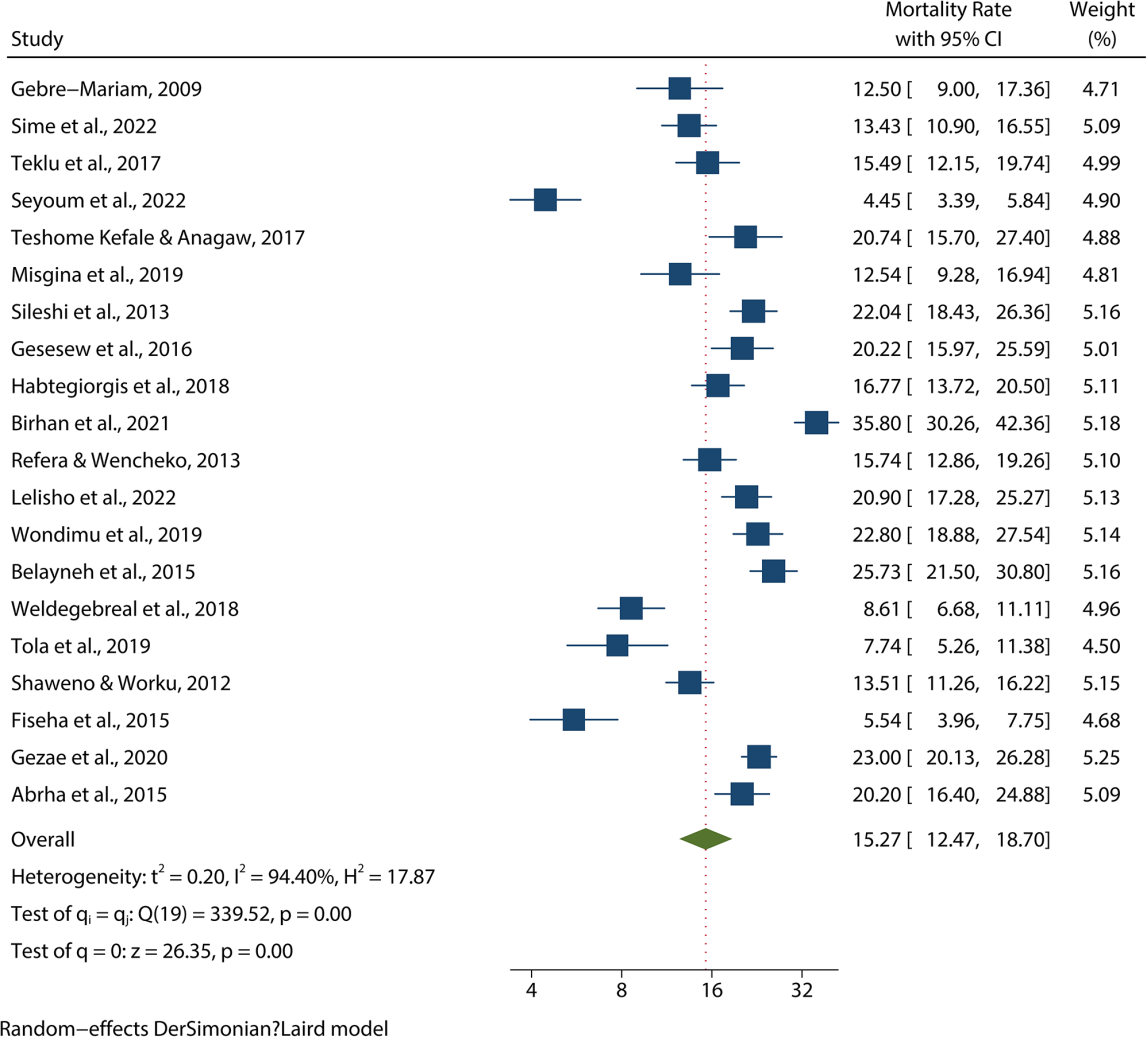
Pooled magintude of mortality among TB/HIV co-infected patients

The overall prevalence of mortality weight among patients with TB-HIV co-infection in Ethiopia is 15.27% (95% CI 12.47–18.70%). There is significant heterogeneity between studies (I²=94.40%, *p* = 0.00). The studies included in the analysis had a wide range of mortality rates, from 4.45 to 35.80%. The lowest reported mortality rate was 4.45% (95% CI: 3.39–5.84) [13], and the highest reported mortality rate was 35.8% (95% CI: 30.26–42.36) [15] (Fig. 2).

### Subgroup analysis

The subgroup analysis based on study setting revealed varying magnitudes of TB/HIV co-infection mortality across different healthcare facility types. Studies conducted in hospitals reported the highest pooled mortality rate of 17.664% (95% CI: 13.749–21.578) based on data from 15 studies. In contrast, studies conducted solely in health centers had a lower pooled mortality of 13.195% (95% CI: 10.877–15.513) from 2 studies. The subgroup involving studies from both hospitals and health centers had a pooled estimate of 14.282% (95% CI: 3.982–24.582) based on 3 studies. When stratified by regional states within Ethiopia, the subgroup analysis highlighted regional variations in TB/HIV co-infection mortality. The highest pooled mortality was observed in the Amhara region at 28.726% (95% CI: 15.247–42.206) from 2 studies. This was followed by Tigray at 20.387% (95% CI: 12.562–28.212) from 3 studies, and Oromia at 19.007% (95% CI: 16.348–21.667) from 4 studies. Relatively lower pooled estimates were seen in Dire Dawa at 14.964% (95% CI: 11.701–18.226) from 2 studies, Southern Ethiopia at 15.395% (95% CI: 7.616–23.174) from 4 studies, Addis Ababa at 10.590% (95% CI: 3.914–17.266) from 3 studies, and Harari at 8.284% (95% CI: 6.536–10.032) from 2 studies. Regarding study design, retrospective studies reported the higher mortality 15.381 (95%CI: 12.441–19.016). Overall, the subgroup analyses suggest that both study setting and geographic region may contribute to the heterogeneity observed in TB/HIV co-infection mortality estimates across the different primary studies included in this systematic review and meta-analysis (Table 2).

**Table 2:** Subgroup Analysis of magintude of mortality among TB/HIV co-infected patients by study setting, study region and study design

Group	No. of studies	Magintude of mortality (%)	[95% conf. interval]
**Study setting**			
Health Center	2	13.195	10.877, 15.513
Hospital & Health center	3	14.282	3.982, 24.582
Hospitals	15	17.664	13.749, 21.578
**Study Regions**			
Addis Ababa	3	10.590	3.914, 17.266
Amhara	2	28.726	15.247, 42.206
Dire Dawa city administration	2	14.964	11.701, 18.226
Harari	2	8.284	6.536, 10.032
Oromia	4	19.007	16.348, 21.667
Southern Ethiopia	4	15.395	7.616, 23.174
Tigray	3	20.387	12.562, 28.212
**Study design**			
Cross-sectional	3	14.488	6.256, 33.554
Retrospective cohort	17	15.381	12.441, 19.016
Overall (%)	20	15.27	12.47, 18.70

### Meta-regression

Significant heterogeneity was detected across the included studies estimating TB/HIV co-infection mortality (I^2 = 95.98%, *p* < 0.001). To investigate potential sources contributing to this substantial heterogeneity, a meta-regression analysis was performed. This explored the impact of various study-level characteristics such as sample size, study region, study setting, study design, and publication year on the mortality estimates. The meta-regression revealed that sample size and study region were significant predictors, together explaining approximately 25% of the observed heterogeneity. Specifically, studies with larger sample sizes reported significantly lower mortality rates (coefficient = -0.0253203, 95% CI: -0.0425264 to -0.0081143, *p* = 0.004), suggesting smaller studies tended to overestimate mortality. Additionally, the regional location where the study was conducted also significantly influenced mortality estimates (*p* = 0.047), with a coefficient of -0.4641044 (95% CI: -2.089261 to -1.161052), indicating certain regions reported lower rates compared to others. However, study setting, study design, and publication year did not appear to be significant contributors to the heterogeneity in this meta-analysis (Table 3). Although accounting for sample size and region reduced the amount of residual heterogeneity, it remained substantial even after these factors were considered (tau^2 = 0.10, I^2 = 74.7%, *p* < 0.001). The variability in our findings may be attributed to unmeasured study characteristics that we didn’t account for in our analysis. These could include differences in patient characteristics, healthcare systems, treatment protocols, outcome measurements, or other subtle factors that vary across studies.

**Table 3:** Meta-regression using sample size, study regions, study setting, publication year and sample designs

Moderating factors	Coefficient	Std. err.	*P* > z	[95% conf. interval]
Sample size	− 0.0253203	0.0087788	0.004	− 0.0425264, − 0.0081143
Study region	− 0.4641044	0.8291768	0.047	-2.089261, -1.161052
Study setting	0.4580355	1.627774	0.778	-2.732343, 3.648414
Publication year	0.5739306	0.578933	0.322	− 0.5607571, 1.708618
Study design	2.303318	4.689691	0.49	-6.888308, 11.49494

### Assessment of publication bias

Publication bias was assessed using Begg’s test, and Egger’s test. Therefore, the Begg’s test gave a z-score of 6.20 with a p-value of 0.0000. The significant p-value suggests there is evidence of substantial publication bias according to Begg’s test. Egger’s test provided a regression intercept of 10.2 with a standard error of 1.654. The p-value for Egger’s test was 0.0000, which is also significant. This indicates that there is small-study effects and evidence of publication bias.

### Factor associated with mortality among TB/HIV co-infected patients

#### Socio-demographic factors

**Fig. 3:**
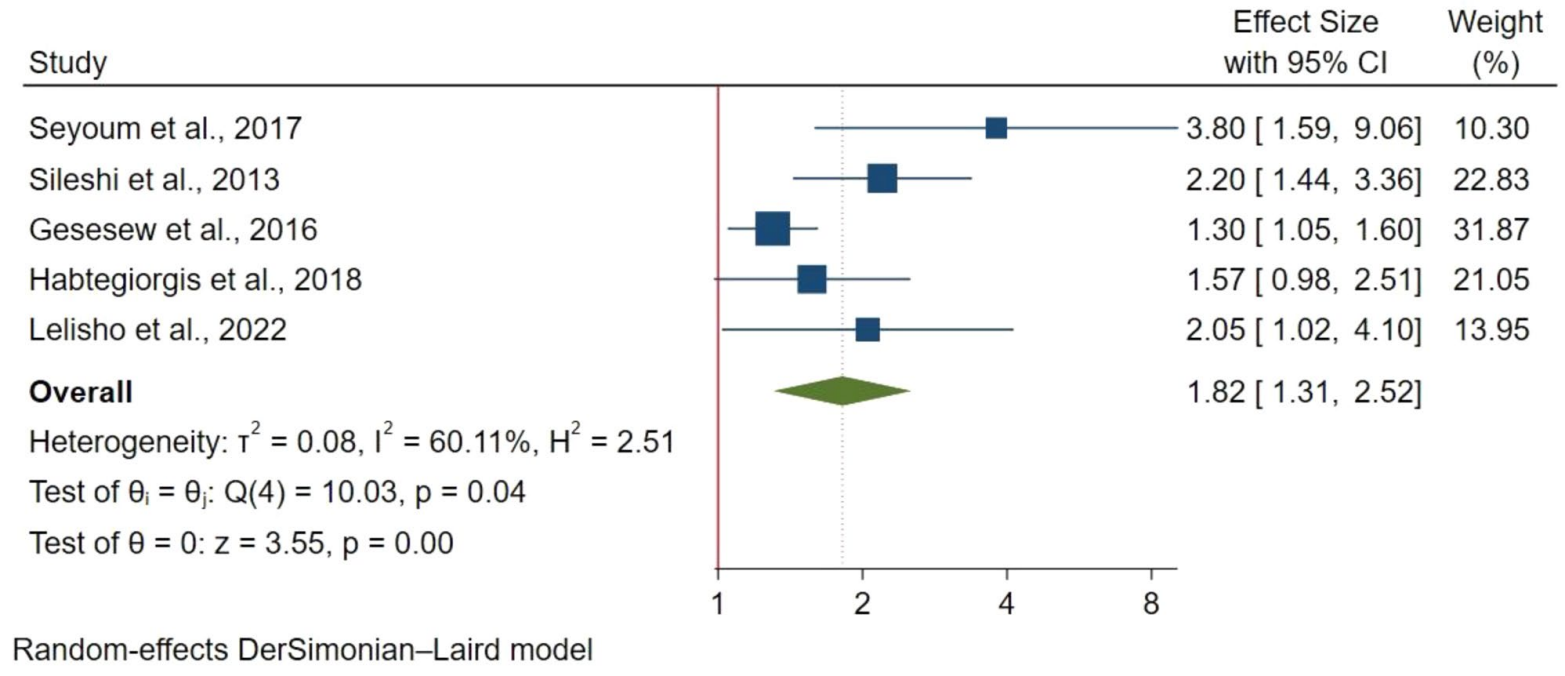
Age > 44 years and mortality in TB/HIV co-infections

Five studies with 1,566 participants identified a significant association between of age above 44 years and TB/HIV coinfection mortality (HR: 1.82; 95% CI: 1.31–2.52) (Fig. 3). However, age 25–34 years (HR: 1.27; 95% CI: 0.66–2.45) and age 35–44 year (HR:1.55; 95% CI: 0.89–2.71) were not significantly associated with mortality.

#### Functional status

**Fig. 4:**
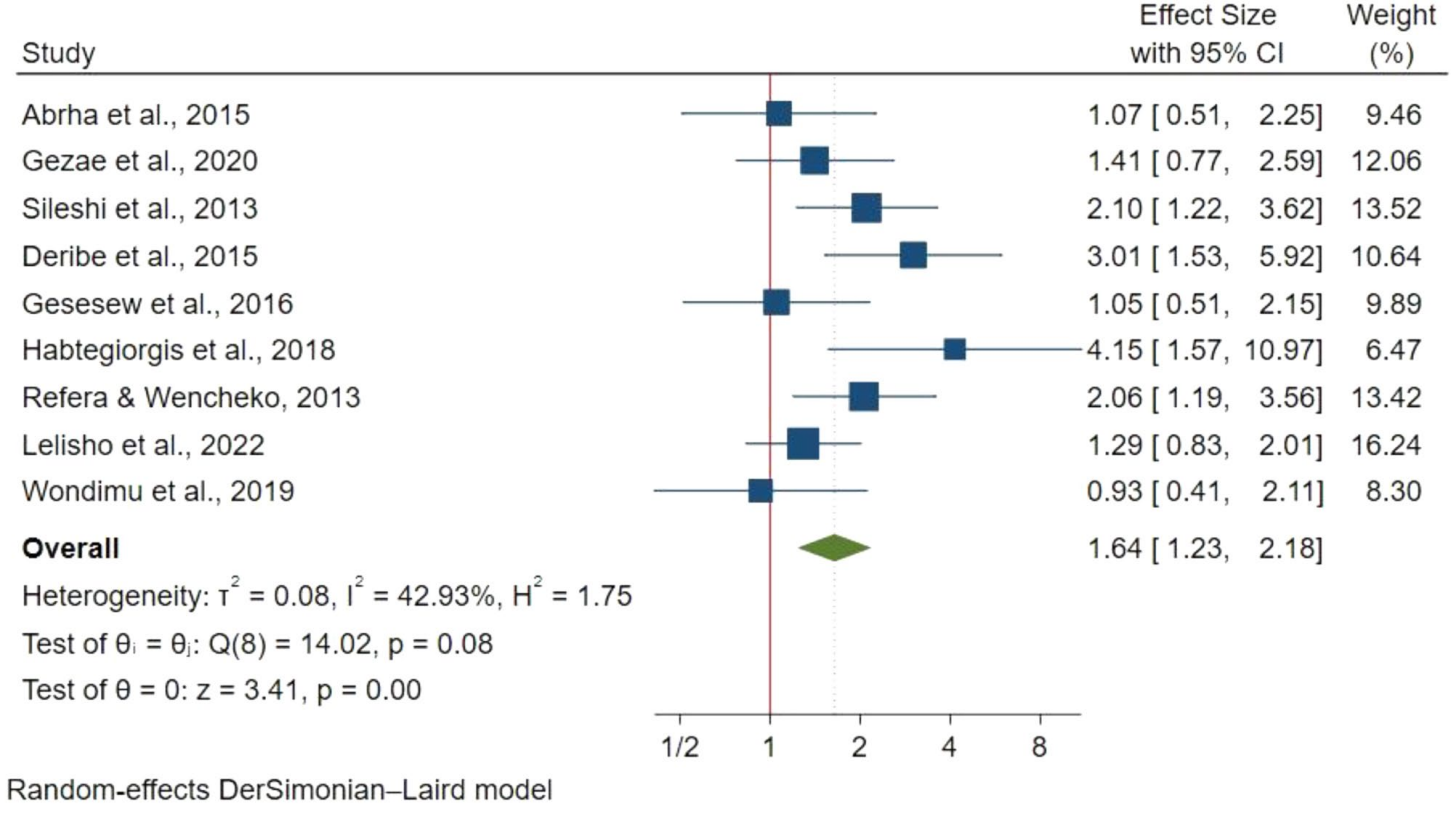
Functional status ambulatory and mortality in TB/HIV co-infections

**Fig. 5:**
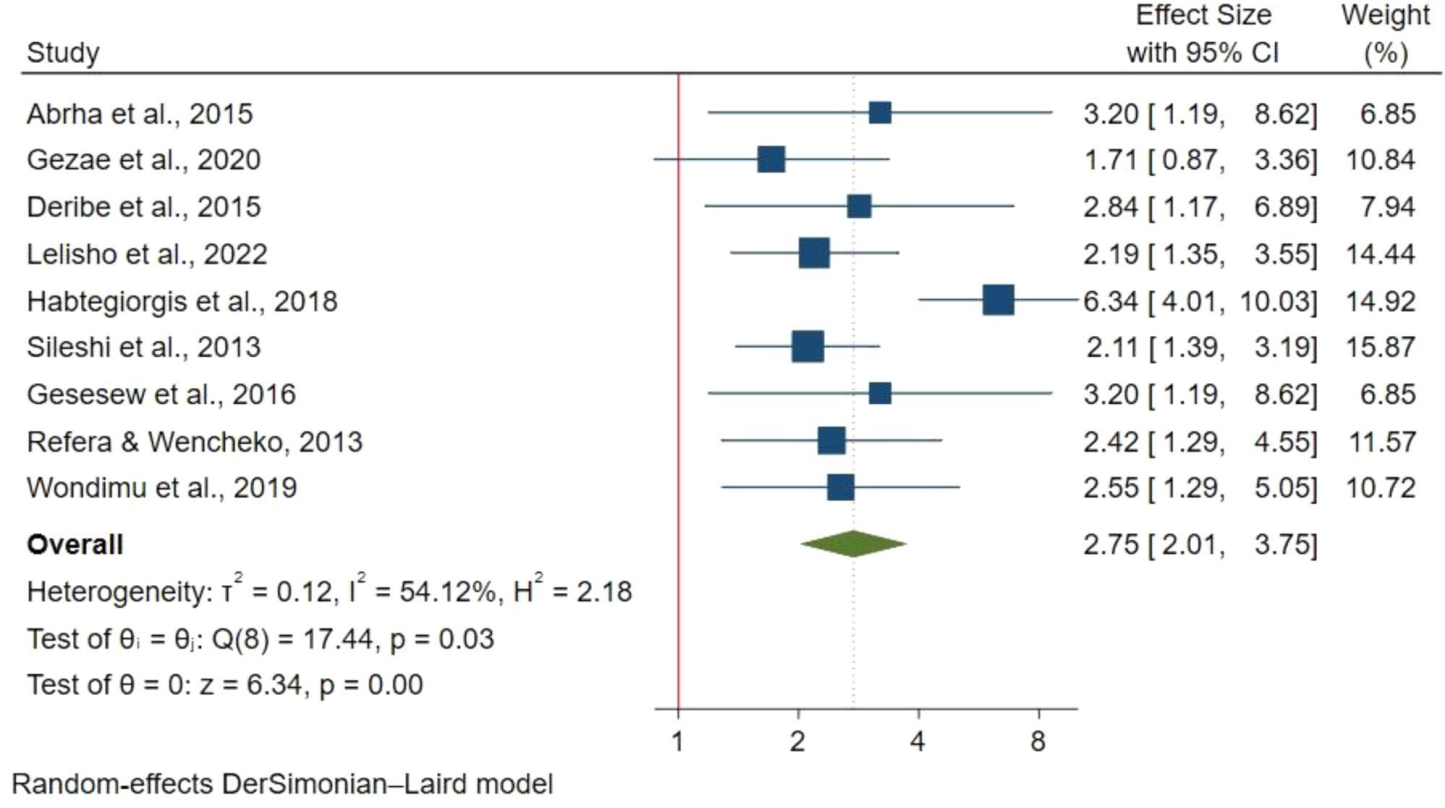
Functional status (bedridden) and mortality in TB/HIV co-infections

Functional status was assessed in 9 studies with 4,112 participants using a fixed effect meta-analysis due to insignificant heterogeneity. Compared to working patients, ambulatory patients had a 64% higher mortality risk (HR: 1.64; 95% CI: 1.23–2.18) (Fig. 4), while bedridden patients had more than twice the risk (HR: 2.75; 95% CI: 2.01–3.75) (Fig. 5).

#### Types of TB

**Fig. 6:**
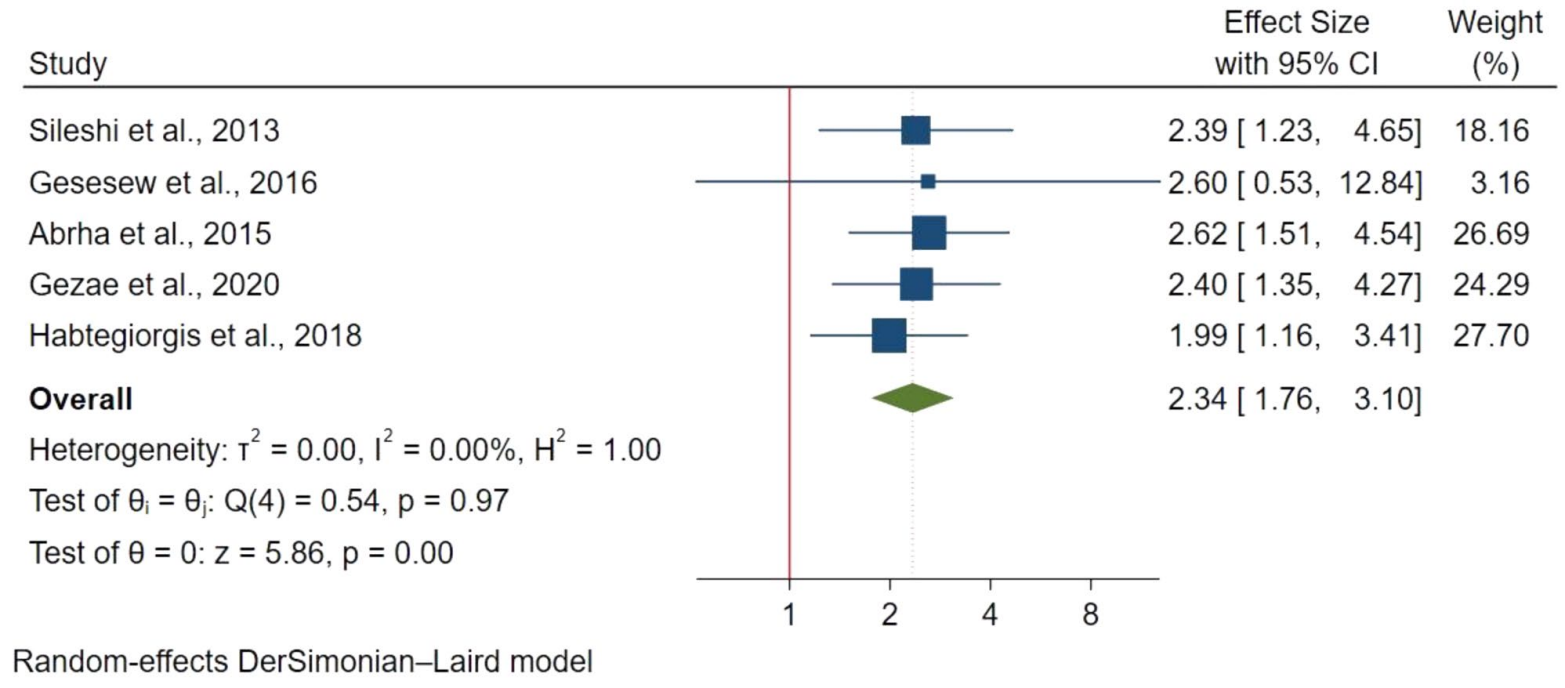
Extra pulmonary Tuberculosis (ETB) and mortality in TB/HIV co-infections

Five studies with 2,017 participants evaluated mortality risk with extra-pulmonary TB compared to pulmonary TB using a fixed effect meta-analysis. Extra-pulmonary TB was associated with a 2.3 times higher risk of mortality (HR: 2.34; 95% CI: 1.76–3.10). (Fig. 6).

#### Co-trimoxazole use

**Fig. 7:**
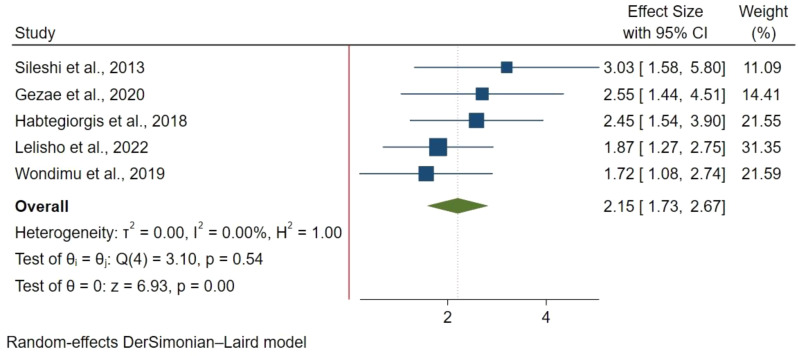
Non-use cotrimoxazole and mortality in TB/HIV co-infections

Lack of co-trimoxazole use was evaluated in 5 studies with 1,998 participants using a fixed effect model. Patients not on co-trimoxazole had twice the mortality risk compared to those on treatment (HR: 2.15; 95% CI: 1.73–2.65) (Fig. 7).

#### WHO staging

**Fig. 8:**
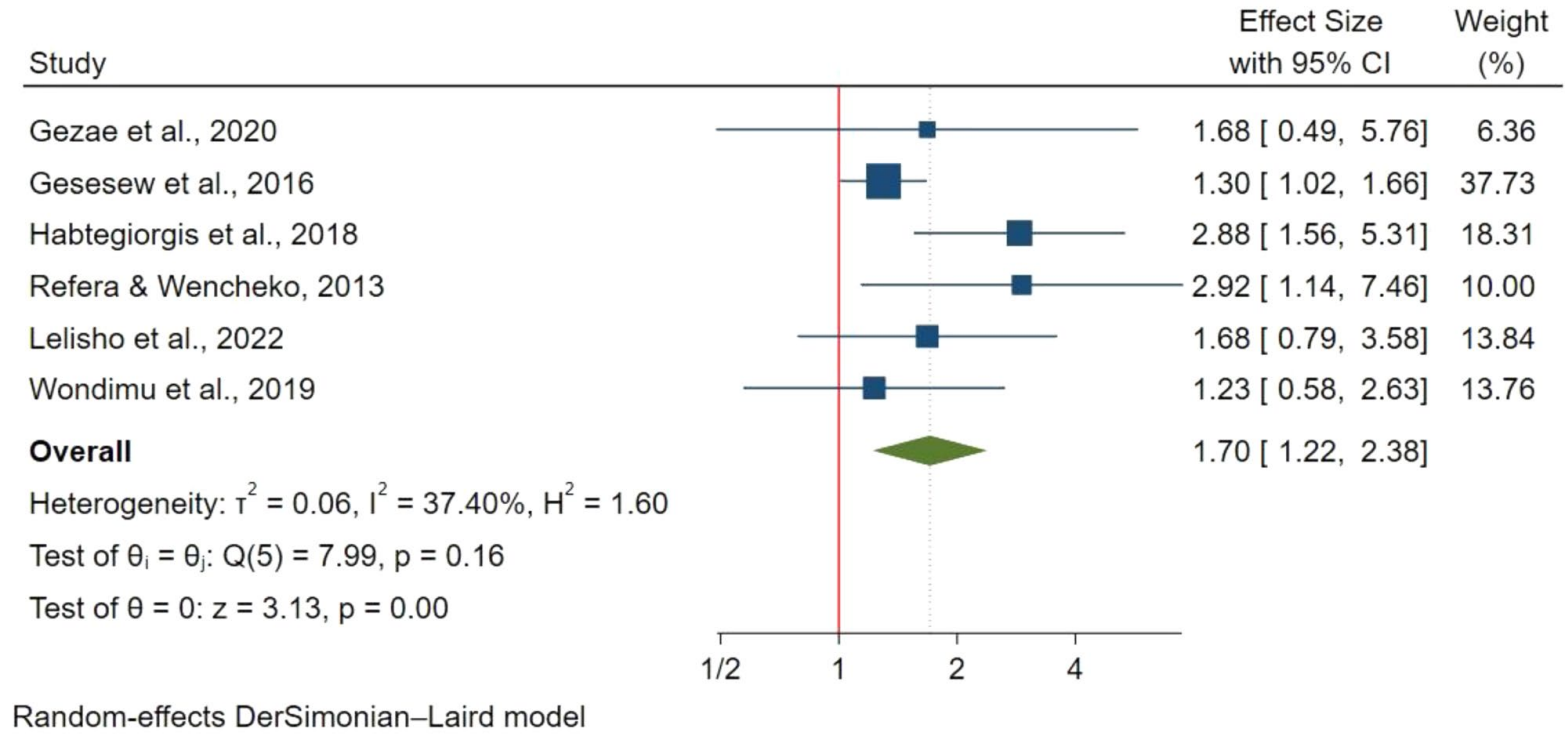
WHO stage III and mortality in TB/HIV co-infections

**Fig. 9:**
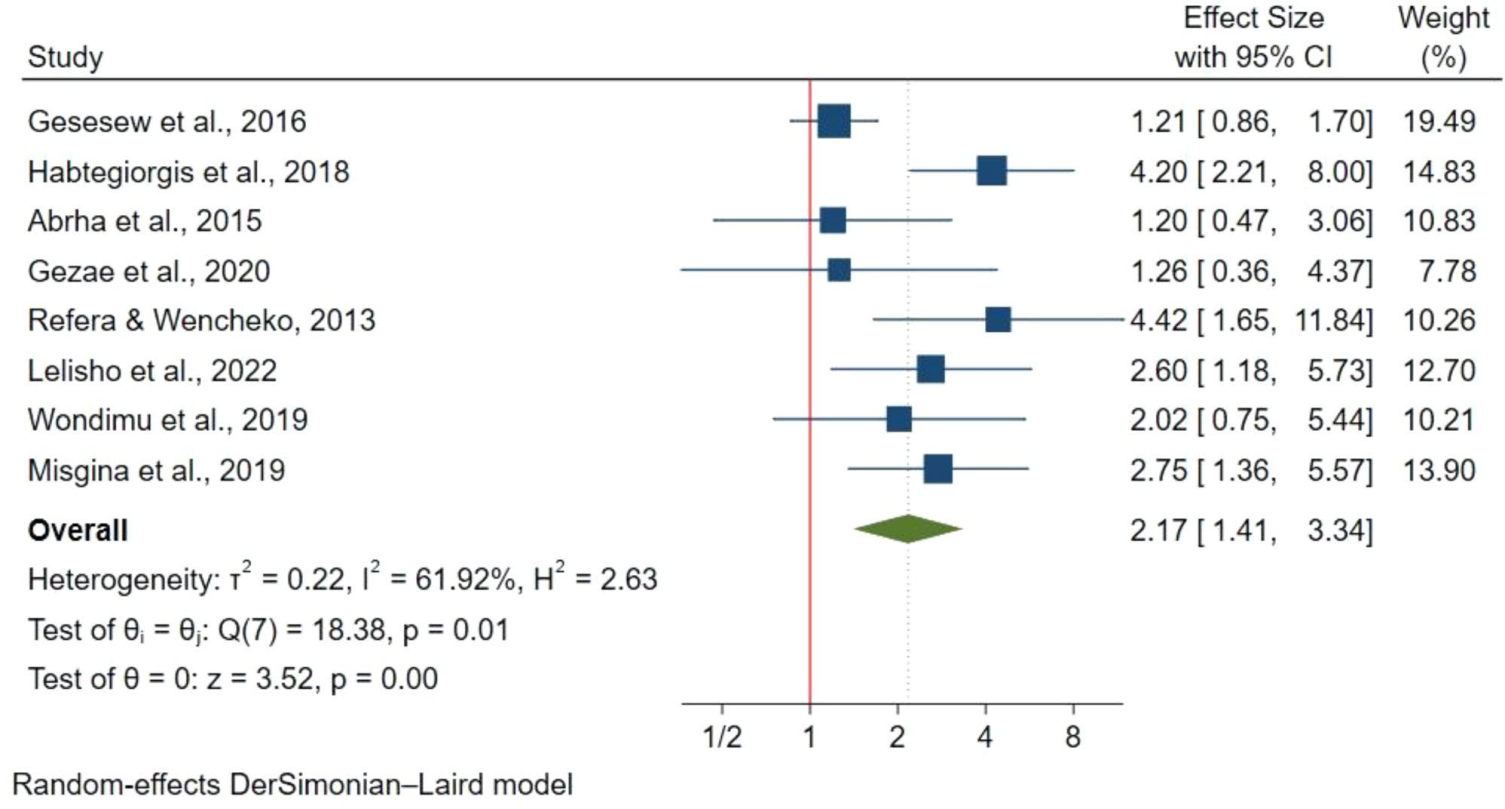
WHO stage IV and mortality in TB/HIV co-infections

WHO stage III was evaluated in 6 studies with 2,543 participants and was associated with a 76% higher mortality risk versus stage I (HR: 1.76; 95% CI: 1.22–2.38) (Fig. 8). Eight studies with 3,112 participants found WHO stage IV had two times higher risk of mortality compared to stage I (HR: 2.17; 95% CI:1.41–3.34) (Fig. 9). However, WHO stage II was assessed in 4 studies with 1,112 participants and was not significantly associated with mortality compared to stage I (HR: 0.58; 95% CI: 0.16–2.12).

#### Opportunistic infections

**Fig. 10:**
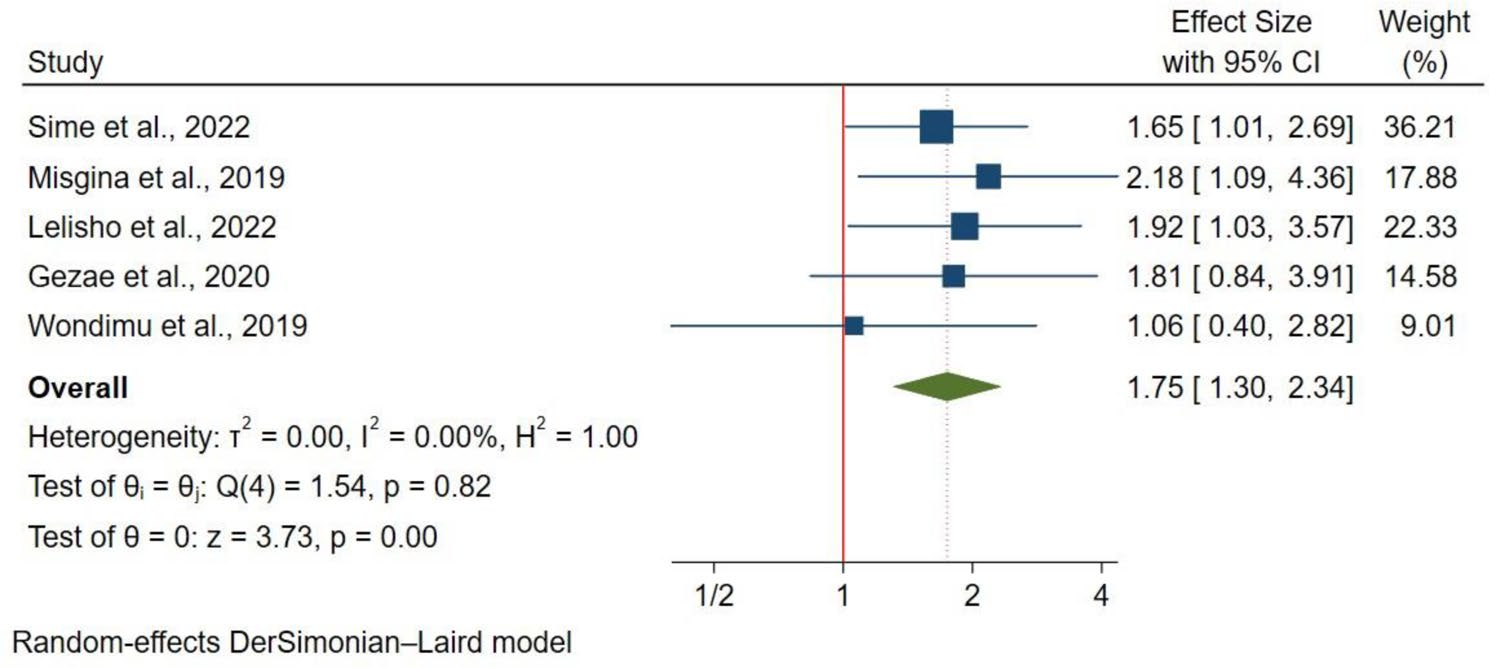
Opportunistic infections and mortality in TB/HIV co-infections

The presence of opportunistic infections was assessed in 4 studies with 1,765 participants using a fixed effect model. Having an OI was associated with a 75% higher mortality risk (HR: 1.75; 95% CI: 1.30–2.34). (Fig. 10).

#### CD4 count

**Fig. 11:**
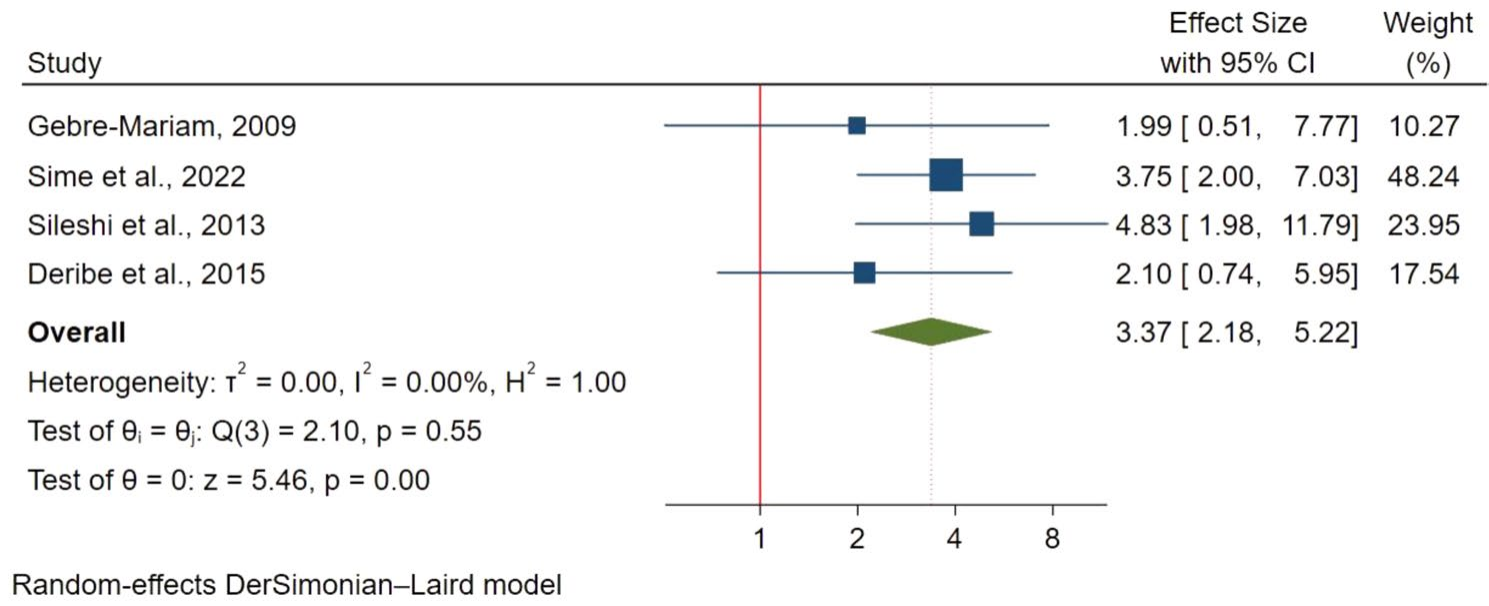
CD4 < 50 cells/mm^3^ and mortality in TB/HIV co-infections

CD4 count < 50 cells/mm3 was evaluated in 4 studies with 1,331 participants and was associated with a 2.6 times higher mortality risk (HR: 3.37; 95% CI: 2.18–5.22) as compared to CD4 count ≥ 200 cells/mm3 (Fig. 11). However, a CD4 count of 50–199 cells/mm3 was not significantly associated with TB-HIV co-infection mortality as compared to ≥ 200 cells/mm3 (HR: 2.49; 95% CI:0.87–7.15).

## Discussion

This systematic review and meta-analysis demonstrated an alarmingly high mortality prevalence of 16.65% (95% CI 12.57%-19.65%) among patients with TB/HIV co-infection in Ethiopia. Several factors were associated with an increased risk of death, including older age, poor functional status, extra-pulmonary TB, advanced WHO stage, opportunistic infections, low CD4 count, and lack of co-trimoxazole prophylaxis.

The observed mortality rate substantially exceeds estimates for HIV mono-infection (15%) [40] in ethiopia or TB alone (4.5%) [41] in Ethiopia, and 8.0% Cameron [42] underscoring the devastating synergistic impact of the dual epidemic. While concerning, this rate is lower than some high HIV/TB burden African countries like Malawi and South Africa [43], where the mortality rate among hospitalized patients with HIV/TB co-infection reached 31% died. It is also higher than the rates reported in India (15.7%) [44], Central/Northern Europe, Southern Europe, and Argentina (7%, 9%, and 11%, respectively) [45]. Hence, when compared to HIV mono-infection, the co-infected population’s mortality rate is noticeably higher, highlighting the devastating synergistic effects of the dual epidemic and pressing the need for focused interventions. The significantly higher mortality associated with co-infection is probably due to the synergistic interaction between HIV and TB. A meta-analysis on HIV/AIDS mortality in Ethiopia reported that most studies (82%) found a mortality rate of 5–15% [46]. Furthermore, a study reported a significantly higher proportion of deaths among HIV-infected TB patients (29.1% versus 15.2%) than in HIV-uninfected TB patients [47] supporting the argument for a synergistic effect. This vulnerable population should be prioritized for interventions to improve timely diagnosis, treatment initiation, retention in care, and clinical management. However, the current mortality report is lower than some African countries’ death rates. For instance, in Tanzania, the mortality rate was 29.1% [47] and in Malaysia, it was 34.9% [48]. Additionally, 64 patients (20.6%) died in Brazil [49]. In contrast, according to a fifteen-year trend study on treatment outcomes among patients with pulmonary smear-positive tuberculosis, a lower death rate ranging from 1.6 to 11.1% was observed [41]. Similarly, in Shanghai municipality, 17.7% of admitted patients died within one year [50]; however, this report was limited to hospitalized patients, who tend to have more advanced disease. Therefore, the findings of this study highlight the critical need for targeted interventions to address the synergistic impact of TB-HIV co-infection on mortality in Ethiopia. Future research should focus on developing and evaluating integrated care models that can improve early diagnosis, treatment initiation, and retention in care for co-infected patients. Longitudinal studies are needed to better understand the long-term outcomes and identify critical time points for intervention.

While older age consistently emerged as a risk factor for higher mortality across multiple studies, the influence of sex and education level yielded contrasting findings. Advancing age may contribute to waning immune function, accumulation of comorbidities, and delays in diagnosis and treatment initiation among elderly patients [51]. However, no significant difference in mortality was observed between males and females, contrary to reports from high TB/HIV burden regions suggesting a higher risk in men [52]. Therefore, future research should focus on understanding the mechanisms behind age-related mortality risk and exploring potential protective factors that could mitigate this risk.

Several markers of advanced HIV disease strongly predicted increased mortality risk, including poor functional status, extra-pulmonary TB, late WHO stage, and severe immunosuppression defined by low CD4 counts. Bedridden patients faced a dramatically elevated risk exceeding 175%, reflecting the limited treatment options and grave prognosis when patients present with profound debilitation. The two-fold higher mortality associated with extra-pulmonary TB aligns with evidence of greater mycobacterial dissemination and heightened disease severity in the setting of HIV co-infection [53]. Similarly, mortality risk increased progressively with advancing WHO stage, mirroring HIV disease progression. Lastly, a steep survival gradient was observed with decreasing CD4 cell counts specially CD4 count of < 50 cells/mm^3^ based on this analysis, reiterating the detrimental impact of severe HIV-mediated immunosuppression and consistent findings were reported from other studies African [11, 47, 54], and other countries [12, 50]. Collectively, these advanced disease markers signal a precipitous decline among co-infected patients, emphasizing the necessity of early therapy initiation. It is imperative that patients with TB and HIV co-infection receive treatment as soon as possible since there is a substantial correlation between advanced HIV disease indicators and a higher risk of death [6]. Healthcare systems should place a high priority on identifying and treating patients with severe immunosuppression, late WHO stage, extra-pulmonary TB, and poor functional status, especially if their CD4 count is less than 50 cells/mm3. These results highlight the critical need for early implementation of antiretroviral and anti-tuberculosis treatments, enhanced access to CD4 testing, and extensive screening programs.

Failure to receive co-trimoxazole prophylaxis was associated with twice the mortality risk compared to treated patients. This finding corroborates data from sub-Saharan Africa, demonstrating over 40% reduced mortality with co-trimoxazole use in TB/HIV that reported co-trimoxazole prevents life-threatening opportunistic infections like pneumocystis pneumonia, which commonly afflict advanced HIV patients [55]. Despite clear survival benefits, co-trimoxazole remains under-prescribed for TB/HIV co-infected populations [56]. Interventions ensuring universal implementation of this low-cost prophylactic regimen could significantly influence mortality reduction. The review’s conclusion emphasizes how crucial it is that patients who are co-infected with HIV and TB receive this care. Given its shown effectiveness in lowering mortality and avoiding potentially opportunistic infections [3, 10, 16], healthcare systems ought to give priority to implementing universal co-trimoxazole prophylaxis for this group. Future studies should concentrate on determining and resolving obstacles to co-trimoxazole prescription and adherence, as well as formulating plans to guarantee the broad, uniform application of this inexpensive, highly effective medication in TB-HIV treatment programs.

Both ambulatory and bedridden functional status conferred substantially higher mortality risk compared to working status, with bedridden patients facing over 175% increased mortality. These findings mirror studies in HIV and TB populations globally, showing incrementally higher mortality as functional status declines [10, 57]. In the context of TB/HIV co-infection, profound functional debilitation likely signals multi-system dysfunction arising from the compounding effects of uncontrolled opportunistic infections, advanced immunosuppression, and end-organ TB involvement [49]. This extremely high-risk population with limited physiologic reserve requires urgent intervention, including intensive support through enhanced screening, rapid diagnosis and treatment initiation, nutritional assistance, and palliative care services.

Compared to pulmonary TB, extra-pulmonary TB doubled the mortality risk in our analysis, consistent with data from other high HIV/TB burden African settings [58]. Disseminated TB, particularly central nervous system and miliary involvement, carries a poorer prognosis in advanced HIV due to the heightened mycobacterial seeding enabled by unchecked immunodeficiency [59]. While some studies in HIV-negative populations show increased mortality with extra-pulmonary TB [60] the detrimental effect is accentuated in the presence of HIV co-infection. The higher mortality risk associated with extra-pulmonary TB in TB-HIV co-infected patients emphasizes the need for improved diagnostic strategies and early detection methods for disseminated TB, particularly in advanced HIV cases [1, 43]. Healthcare providers should maintain a high index of suspicion for extra-pulmonary TB manifestations in this population to ensure timely intervention. Optimal timing of ART to support immune reconstitution and treatment of drug-resistant TB strains are critical in this population.

### Limitations and recommendations

Our review has certain limitations to consider. Most included studies were facility-based cohorts, which may overestimate mortality compared to community populations. Publication bias may also skew findings toward significant associations. There was heterogeneity between studies that may be attributed to unmeasured study characteristics that were not accounted for in our analysis, and studies used to identify predictors were small for some predictors which may affect generalizations.

## Conclusion and recommendations

This systematic review and meta-analysis demonstrated a high mortality prevalence of 16.65% among TB/HIV co-infected patients, substantially exceeding estimates for HIV or TB mono-infection in Ethiopia. Older age, poor functional status, advanced immunodeficiency, and lack of co-trimoxazole prophylaxis were key factors associated with mortality risk. To reduce the high mortality in this vulnerable population, interventions should focus on strengthening integrated TB/HIV services, ensuring prompt diagnosis and early initiation of therapy, strategic timing of ART, optimal prophylaxis and management of co-morbidities, and improving retention along the care cascade. A patient-centered approach with sustained clinical and adherence support is essential. At the health systems level, capacity building and adequate resourcing for TB/HIV care, uninterrupted drug supplies, and better data systems are needed. Addressing the complex factors driving mortality will require a multisector response involving stakeholders across programs, levels, and partners.

## Data Availability

All data analyzed during this study are included in this published article.

## Abbreviations

AIDS: Acquired immunodeficiency syndrome
ART: Antiretroviral Therapy
ETB: Extra pulmonary Tuberculosis
TB: Tuberculosis
PRISMA: Preferred Reporting Items for Systematic Reviews and Meta-Analyses
HR: Hazard Ratio
MeSH: Medical Subject Headings
HIV: Human Immunodeficiency Virus
